# The role of orthobiologics in hip preservation surgery

**DOI:** 10.1093/jhps/hnv042

**Published:** 2015-08-27

**Authors:** Zeiad Alshameeri, Andrew McCaskie

**Affiliations:** 1. Health Education East of England, 2-4 victoria house, Cambridge, CB21 5XB; 2. Division of Trauma and Orthopaedic Surgery, Department of Surgery, University of Cambridge and; 3. Arthritis Research UK Tissue Engineering Centre

## Abstract

The potential regenerative role of different orthobiologics is becoming more recognized for the treatment of chronic and degenerative musculoskeletal conditions. Over the last few years there has been an increasing number of publications on cell therapy and other orthobiologics for the treatment of avascular necrosis of the femoral head and other hip conditions with promising short–term clinical results. In this article, we have used a systematic search of the literature to identify potentially relevant topics on orthobiologics and then selected those most applicable to hip preservation surgery. We identified several innovative strategies and present a summary of the currently available evidence on their potential role in hip preservation surgery. For many of these treatment strategies there was a lack of clinical evidence and therefore we suggest that there is a need for comparative studies in this field.

## INTRODUCTION

Orthobiologics have been defined for a non-specialist audience as ‘substances that orthopaedic surgeons use to help injuries heal more quickly’ that are ‘made from substances naturally found in the body’ which can be used in problems such as ‘the healing of fractured bones, injured muscles, tendons and ligaments’ [1]. The term ‘Orthobiologic’ can therefore be considered to refer to a biologically derived material used in the regeneration and repair of musculoskeletal tissues. This would also encompass osteoconductive substances that provide the conductive medium to facilitate the in-growth and expansion of the normal tissue [2, 3].

A number of studies reporting on the clinical outcomes of incorporating these substances in different therapies identified a spectrum of potential clinical applications [4]. Initially this was in the field of trauma and reconstructive surgery [2] but there is now a growing recognition for a potential role in chronic and degenerative musculoskeletal conditions [3–5]. Their utilization in isolation or in conjunction with traditional surgical procedures in the prevention or the treatment of early degenerative conditions has thus far yielded promising short-term clinical results [6–8].

Regenerative medicine is an expanding clinical area which encompasses both cell and cell-free approaches to treatment, and can make use of orthobiologics. It is therefore no surprise that regenerative and orthobiologic treatments might be considered in hip preservation. Furthermore, minimally invasive hip surgery in general and hip arthroscopy specifically have facilitated early interventions and treatment for multiple hip conditions including early stages of osteoarthritis in young patients [9, 10]. This has also brought different types of ‘orthobiologics’ into the spot light of hip preservation surgery.

Therefore our aim was to conduct a systematic literature search looking for articles reporting the use and outcome of orthobiologic approaches in hip preservation surgery.

## LITERATURE SEARCH METHOD

A systematic literature search was conducted using the EMBASE (between 1974 and November 2014) and PubMed data bases for entries on orthobiologics and hip preservation surgery. Initially we searched for any entries containing the key ward ‘orthobiologics’. There were only 41, and none related to ‘hip preservation surgery’. However, these were reviewed and used to identify the common ‘orthobiologic’ substances used in surgery. The resulting terms were then used for the main literature search. The first search (search 1) was conducted for records containing any of the terms relating to ‘orthobiologics’ (Table I). The ‘explode’ function on EMBASE search engine was also used to include any articles containing any other terms relating to the key words used in the search. The ‘OR’ function (in PubMed and EMBASE databases search engines) was used to compose a list of all the hits containing at least one of the search key wards used in search one. A second search was carried out with the key words ‘hip’ OR ‘Femoral head’ (search 2). The Explode function was also used for this search in the EMBASE data base.

**Table I.** The terminologies used for database searches

Search category	Keywords
Search1	‘stem cell’ OR ‘BMP’ OR ‘PRP’ OR ‘thrombocyte rich plasma’ OR ‘HA’ OR ‘bone marrow stem cell’ OR ‘hematopoietic stem cell’ OR ‘peripheral blood stem cell’ OR ‘mesenchymal’ OR ‘mesenchymal stroma cell’ OR ‘mesenchymal stem cell’ OR ‘cartilage transplantation’ OR ‘cartilage cell’ or ‘cell transplantation’ OR ‘bone graft’ OR ‘bone substitute’ OR ‘autologous chondrocyte implantation’ OR ‘ACI’ or ‘matrix-induced chondrocyte implantation’ OR ‘MACI’ OR ‘chondrocyte implant’.
Search 2	‘hip’ OR ‘femoral head’

**Fig. 1.**
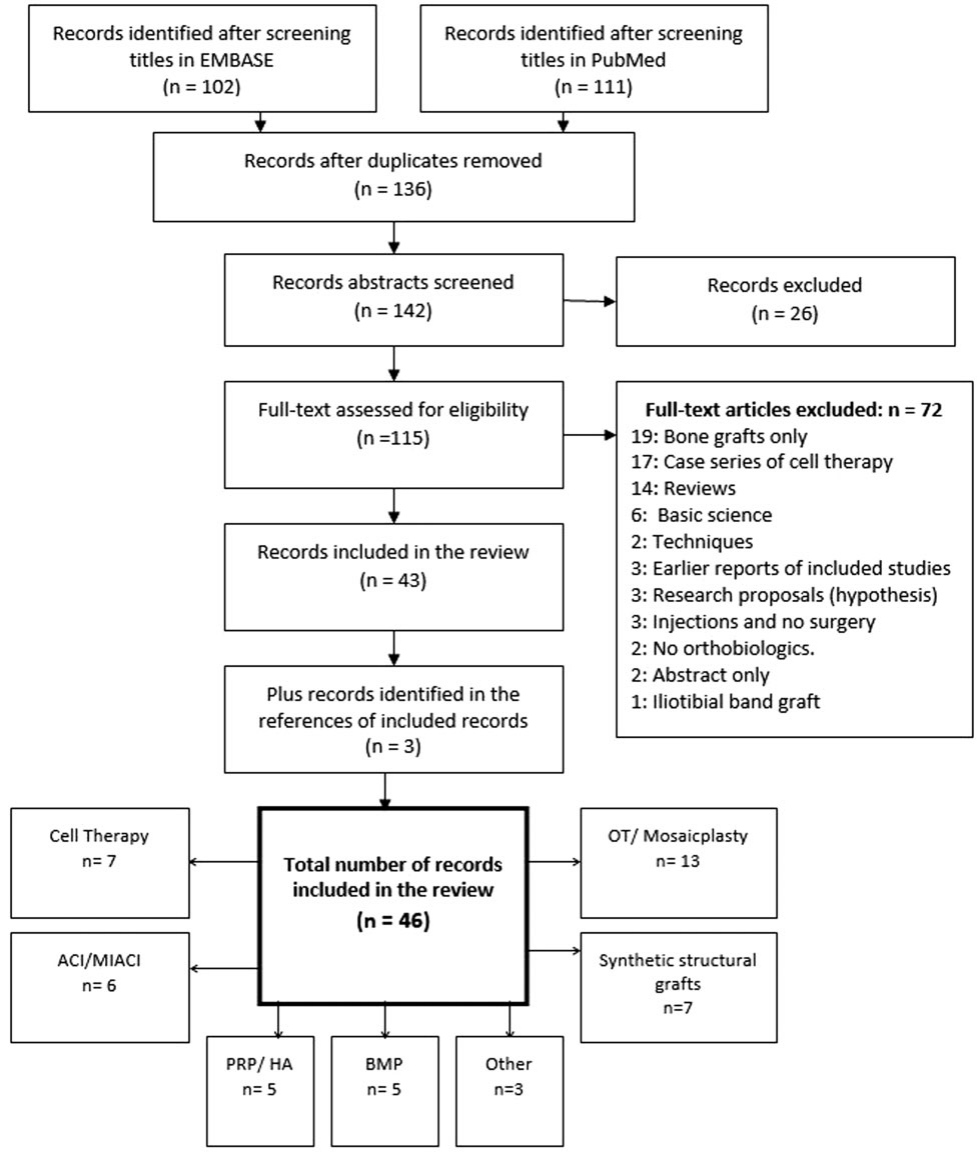
The inclusion and reviewing process of the articles. Some of the final included articles incorporate more than one orthobiological substance.

The results of Search 1 and Search 2 were combined with the function ‘AND’ in both data bases. This yielded a list of hits (in each data base) containing at least one of the keywords used in search 1 ‘AND’ at least one of the keywords used search 2. Only entries in the English language and in human subjects were selected, yielding a total of 2681 hits in EMBASE and 1473 hits in PubMed. The titles of these entries were screened in order to identify relevant articles. For further inclusion, our criteria required titles to relate broadly to the use of orthobiologics and any form of hip surgery. If the title was ambiguous then the abstract was also read to decide on the relevance of the article. Subsequently, the abstracts of all the identified selected titles were read to exclude any articles that did not satisfy our selection criteria. If the abstract was not clear, the full text was retrieved. This was followed by retrieving the full text of all the identified relevant articles for review. Animal or *in vitro* studies were excluded. Articles on the use of human bone graft in isolation were excluded unless synthetic bone graft substitutes were used. We only included comparative control studies such as case control, cohort or randomized control studies. Case series and case reports were only included if no (or small number of) comparative studies were found. We also cross referenced all identified articles and systemic reviews to make sure no relevant articles were missed by the data base searches, Fig. 1. If more than one article was found on the same study, then only the latest article with the longest follow-up was included.

## RESULTS

A large number of articles on cell therapy treatment for Avascular Necrosis of the Femoral Head (AVNFH) were found. Therefore only clinical comparative control studies, including five randomized control trials (RCTs), were included in the review [11–18] (Table II). Case series reports on cell therapy were only referred to in the text if they had addressed a significant development in cell therapy not covered by the control studies. With regards to the rest of orthobiologics, there were a very small number of control studies [19–22] (Table III) and therefore we included all case series and case reports found during the literature search [23–56]. Although we have referred to all these publications, only the main articles are summarized in this review.

**Table II.** Comparative studies on the use of cell therapy for the treatment of AVNFH

Study type	Cohort of patients	Procedure	Source of cells	FU and Outcome
**Mao *et al*. (2014): RCT study** comparing the effect of mechanical support and targeted intra-arterial infusion of PBSCs versus mechanical support alone on the progression of AVNFH.	– Hips with AVNFH. – ARCO Sages 1 to 3. – **Tr Gp;** 48 hips (30 patients) received mechanical support and infusion of PBSC. – **Ct Gp**: 41 hips (25 patients) mechanical support only.	– CD carried out with 10-mm-diameter trephine. – Cylindrical shape porous tantalum rod inserted into the necrotic area. – Tr Gp: 7-day post-op, PBSCs infused via medial circumflex femoral artery – Hips in the control group were not given any placebo.	Autologous PBSCs harvested by apheresis from a peripheral blood sample after a subcutaneous injections of 10 μg/kg of G-CSF for 4 days. Average of 2.47× 10^8^ mononuclear cells containing 1.71× 10^6^ CD34+ were administered into each hip.	**FU:** 36 months for all cases. **HHS:** mean improvement in the Tr Gp was significantly higher than the Cr Gp (88.1 vs. 78.4 respectively; *P*= 0.003). **Radiological progression:** – Progression observed in 4 (8.3%) in the Tr Gp versus 13 (31.7%) in the Cr GP (*P*= 0.005). – No significant difference in collapse rate between the two groups. **Conversion to THA:** – 3 (6.25%) in the Tr Gp versus 9 (21.95%) in the Cr Gp (*P*= 0.031). – Higher survival rate in the Tr Gp at 36 months (*P*= 0.025).

**Ma *et al*. (2014): RCT study** comparing the effect of BMMCs combined with CD and Bone graft versus CD and graft for the treatment of AVNFH.	– Hips with AVNFH. – Ficat stage I to III. – **Tr Gp:** 25 (21 patients) hips CD and autologous bone graft with BMMCs – **Ct Gp:** 24 (18 patients) hips CD and autologous bone graft.	– CD with 10-mm diameter trephine. – Necrotic area curetted. – A cylinder of bone from the femoral neck and head was used for bone marrow grafting. – For the Tr Gp, the cells were added to the bone graft before implantation.	Autologous bone marrow aspirate from the anterior superior iliac spine. Cells were concentrated. 1 ml containing 3× 10^9^ nucleated cells were added onto the porous cylindrical bone.	**FU:** 24 months for all cases. **Pain Visual Analogue Score (VAS):** – Decreased in both groups but more significantly in the Tr Gp. – Ct Gp mean pain VAS decreased from 35.21 to 26.46 (*P*= 0.007). – Tr Gp mean pain VAS decreased from 35.58 to 16.92 (*P*< 0.001). **WOMAC and Lequesne index** improved in both groups but more significantly in the treatment group: – Tr Gp mean Lequesne index improved from 9.58 to 5.83 (*P*< 0.001) – Tr Gp mean WOMAC decreased from 27.77 to 14.81 (*P*= 0.001). **Radiological progression:** – Ct Gp; 8 hips (33.3%) progressed and 4 needed THA. – Tr Gp: 2 hips(8%) progressed and both needed THA.
**Rastogi *et al.* (2013): cohort control study** comparing the effectiveness of isolated and concentrated BMMCs versus unprocessed bone marrow installation for AVNFH	– Hips with AVNFH – ARCO stages I, II and III. – **Tr Gp;** 30 hips received CD and isolated BMMCs instillation. – **Ct Gp;** 30 hips received CD and unprocessed bone marrow instillation.	– Two core decompression tracts made through lateral femoral cortex into the necrotic area using 4.5-mm reamer. – Tr Gp: isolated BMMCs (1.1 × 10^8^ cells) injected into the necrotic zone. – Ct Gp: unprocessed marrow (30–50 ml) injected into the necrotic lesion. – CD canal plugged with gel foam after installation of cells and bone marrow	60–70 ml of bone marrow aspirated from the iliac crest at the beginning of the procedure – For Tr Gp: marrow aspirate was processed to separate and concentrate BMMCs to a final volume of 5 ml containing approximately 1.1 × 10^8^ cells. – Ct Gp: bone marrow aspirate not processed.	**FU:** average 24 months (22–28 months). **HHS:** the mean improvement was 31.85 versus 19.72 points for Tr Gp and Cr Gp, respectively: no statistically significant difference between groups. **Conversion to THA:** 3 (10%) hips in the Ct Gp and none in Tr Gp. **Radiological progression:** 4 (13.3%) hips in Ct Gp and none in the Tr Gp progressed to advanced stages. **Necrotic lesion size:** decreased in both groups but more so in the Tr Gp (*P* = 0.03).

**Liu *et al*. (2013): case control study (**age and gender matched) comparing implantation of nano- hydroxyapatite/ polyamide bone filler with or without BMMCs.	– Hips with AVNFH. – ARCO stage IIB and IIC; – **Tr Gp:** 26 (17 patients) hips received CD, curette of necrotic area + nano- hydroxyapatite/ polyamide bone filler with BMMCs. – **Ct Gp;** 27 (17 patients) hips received CD, curette of necrotic area + nano- hydroxyapatite/ polyamide bone filler without BMMCs.	– CD with 10-mm drill. – Necrotic area decompressed with expanding reamers and curetted. – Ct Gp: granular porous nano-hydroxyapatite/polyamide 66 composite bone filling material was implanted in the bone tunnel. – Tr Gp: the Granular porous nano-hydroxyapatite/polyamide composite bone filling material was soaked in the concentrated BMMCs solution and implanted in the bone tunnel.	150–200 ml bone marrow aspirated from the posterior superior iliac spine of patients in the BMMC group. BMMCs were isolated and purified to 31.4 ± 4.8 × 10^6^ cells/ml.	**FU:** –Tr Gp; average 24.9(18–32) months. –Tr Gp: average 26.7 (12–40) months. **HHS:** increased in both groups (*P < *0.05) but significantly greater in Tr Gp (a mean increase of 28.6 versus 18.4%, *P* < 0.001). **Pain VAS:** decreased in both groups but significantly more in Tr Gp: (a mean decrease of − 66.3 versus − 51.7%, (*P* < 0.001). **Radiological success rate (no femoral head collapse or onset of OA):** higher in the Tr Gp group; 78.6% versus 40.7% (*P* = 0.004) **Clinical success rate (HHS > 80):** higher in the Tr Gp, 75.4 versus 37.0% in the Ct Gp. **Collapse or aggravated collapse:** –Tr Gp; 21.4% (6/28) and 4 (14.3%) converted to THA. –Ct Gp; 59.3% (16/27) and 5 (18.5%) converted to THA.
**Sen *et al*. (2012): RCT study** comparing CD alone versus CD and autologous BMMCs	– Hip with AVNFH. – ARCO stage I–II. – **Tr Gp:** 26 hips received CD and BMMCs. – **Ct Gp:** 25 hips received CD only.	– Multiple CD through the lateral cortex of femur (3 cores of 4 mm in diameter) for both groups. – Tr Gp received BMMC installation into the opening of the core tract and the lateral cortex opening sealed with bone wax.	120–180 ml of bone marrow aspirated from the posterior superior iliac spine. This was processed to obtain 2 ml of BMMCs concentrate (5 × 10^8^) and installed during the same surgical procedure.	**FU:** 24 months for all cases **HSS:** mean improvement at 24 months; 11.67 points versus 16.23 points for Ct Gp and Tr Gp, respectively, but no statistical significance observed between the groups **Radiological changes:** no significant difference in the radiological changes (MRI and x-ray) between both groups.

**Zhao *et al*. (2012): RCT study c**omparing *ex vivo* expanded BMMCs and CD versus CD alone in treating AVNFH.	– Hips with ARCO stage 1 C and 2 C AVNFH – **Tr Gp** (BMMC): 53 hips (50 patients). – **Ct Gp:** (CD) 51 hips in 50 patients (7 hips lost to FU)	– CD and debridement of necrotic lesion – 10 ml of subtrochanteric bone marrow aspirated and sent for processing and culture. – Tr Gp: 2 × 10^6^ cells (in 2 ml) injected into the necrotic area 2 week after core decompression.	10 ml of bone marrow aspirated from the sub-trochanteric region sent for *ex vivo* culture and expansion.	**FU:** 60 months for all cases **HHS:** – Tr Gp had a greater improvement in HSS in all stages of the disease more than Ct Gp (*P* < 0.05). – Ct Gp had no HSS improvement in stage 1C**.** **Disease radiological progression:** – Tr Gp: 2 (3.8%) hips. – Ct Gp: 10 (22.7%) (*P* < 0.05). **Necrotic lesion size**: significantly decrease in size In Tr Gp compared with the Ct Gp in all stages. **THA conversion**: 5 (11.4%) Ct Gp versus 0 hips Tr Gp.

**Gangji *et al*. (2011): RCT study** comparing the efficacy of CD + BMMC versus CD alone	– Hips with AVNFH – ARCO stage 1 or 2. – **Tr Gp:** 13 hips received CD and BMMCs. – **Ct Gp:** 11 hips received CD only	– CD with 3-mm trephine for both groups. – Tr Gp: autologous bone marrow cell implantation into the necrotic zone (bone marrow graft group).	400-ml autologous marrow aspirated from the anterior iliac crest. BMMCs were sorted and concentrated to a mean final volume of 51 ml containing a mean number of leukocytes 2.0 ± 0.3 × 10^9^.	**FU:** 60 months. **Clinical outcome: s**ymptoms significantly better in the Tr Gp versus Ct Gp according to pain VAS (*P* = 0.009) and Lequesne index (*P* = 0.030). No difference in the total WOMAC score (*P* = 0.091). **Necrotic lesion:** decreased in volume in both groups but more so in the Tr Gp (*P* = 0.066) **Progression to ARCO stage 3 (failure):** 8 (72.7%) in the Ct Gp versus 3 (23.1%) in the Tr Gp (p = 0.038). **Time to failure:** quicker failure rate in Ct Gp over 60 months (*P* = 0.008). **Conversion to THA:** 3 (27.3%) in the Ct Gp versus 2 (15.3%) in the Tr Gp. Type of treatment did not delay THA (*P* = 0.42)

**Yamasaki (2010) *et al*.: case control study** comparing the efficacy of IP-CHA graft with or without BMMCs for the treatment of AVNFH	– Hips with AVNFH. – JOC stages 1 to 3A. – **Tr Gp:** 30 hips (22 patients): – **Ct Gp:** 9 hips (8 patients).	– 10-mm burr used for CD tunnel. – Tr Gp; BMMCs were seeded onto interconnected Porous calcium hydroxyapatite (IP-CHA) cylinder. This was then inserted into the CD tunnel. – Cr Gp: cell-free IP-CHA cylinder inserted onto the tunnel.	700 ml of bone-marrow aspirated from the Iliac crest. Cells separated and concentrated into a 40 ml sample of BMMCs containing ∼ 1 × 10^9^ cells.	**FU:** average 29 months (19–45). **Mean Merle d’Aubigné and Postel score:** – Tr Gp: increased from 14.7 to 17.0 points. – Ct Gp: reduced from 15.2 to 14.2 points **Mean pain score:** Tr Gp: improved from 4.2 to 5.5. Ct Gp: three hips needed THA. **Radiological progression:** – No radiological Progression: 17 (56.7%) Tr Gp versus 0 Ct Gp. – Less than 2 mm collapse: 10 (33.3%) Tr Gp, versus 3 (33.3%) Ct Gp. –Greater than 2 mm collapse: 3(10) Tr Gp versus 6 (66.6%) Ct Gp. **THA conversion:** – Tr Gp: 1 (3.3%) hip converted to THA – Ct Gp: 3 (3.3%) hips converted THA

**Table III.** Comparative studies on other orthobiologics in hip preservation surgery

Study type	Cohort of patients	Procedure	FU and outcome
***‘*rhBMP-2’ Sun *et al*. (2014): cohort study** comparing artificial bone (Novobone) with or without rhBMP-2 for the treatment of AVNFH	– Hips with AVNFH. – ARCO stages IIB to IIIA. – Different aetiology. – **Tr Gp:** 33 hips received bone graft and rhBMP2. – **Ct Gp:** 39 hips received bone graft only.	– ‘Light Bulb’ procedure: a bone window made at the femoral head-neck junction. – Necrotic bone debrided with a drill and a curette. – Autologous iliac cancellous bone and artificial bone (NovoBone) were impacted in necrotic cavity. – Tr Gp: received bone graft and 4 mg of rhBMP-2 – CT Gp: received bone graft only.	**FU:** average 6.1 years (5-7.67). **Mean HHS:** – Tr Gp increased from 68.16 to 82.36; (69.7 % with HHS > 80 points). – Cr Gp increased from 67.26 to 78.96; (64.1% with HSS > 80 points). **Radiological outcome:** ‘The roundness and complete repair of the femoral head’ was observed in 34 (94.4%) of hips with HHS > 90 but no distinction made between the two treatment groups.

**‘PRP’ Redmond *et al.* (2014): cohort study** assessing the efficacy of intraoperative PRP interarticular injection on the outcomes of hip arthroscopy for labral tear treatment.	– Patients with labral tears undergoing hip arthroscopy. – **Tr Gp:** 91 patients received intra-articular PRP at the end of the procedure. – **Ct Gp:** 180 patients received 0.25% bupivacaine at the end of procedure.	– Hip arthroscopy	**FU:** minimum 2 years. **Pain score:** higher in Tr Gp 2 years post surgery (3.4 versus 2.5, *P* = 0.005). **MHHS at 2 years**: lower in the Tr Gp (78.6 versus 82.6, *P* = 0.049). **HOSADL:** no significant difference between groups. **HOSSSS:** no significant difference between groups. **NAHS:** no significant difference between groups. **Conversion to THA**: no significant difference between groups. **Revision Hip arthroscopy:** no significant difference between groups.
**‘Bone substitute’ Yang *et al*. (2014): case control study** comparing the use of (n-HA/PA66) rod and a porous bioglass bone graft versus autologous cancellous bone graft following CD for AVNFH	– Hips with AVNFH. – Steinberg stages I, II, or IIIA; – **Ct Gp:** 46 hips received CD + autologous cancellous bone graft. – **Tr Gp:** 38 hips received CD and implantation of a n-HA/PA66 rod + resorbable bioglass bone (NovaBone) graft	– CD with 8 mm cannulated drill. – Necrotic area debridement with a curette (Ct Gp) and expandable reamer instrument (Tr Gp). – Ct Gp: cancellous bone from the intertrochanteric region was loosely placed into the canal. – Tr Gp: the cuboids-shaped NovaBone was press-fit into the necrotic area via the canal and the n-HA/PA66 rod was inserted into the CD channel.	**FU:** average 2 years. – Tr Gp: mean follow-up of (21.78 ± 8.46) months. – Ct Gp: mean follow-up of (23.24 ± 9.32) months. **HSS:** mean HSS significantly improved in both groups: Mean HSS improvement in Tr Gp was greater than the Ct Gp: (27.19 versus 15.58 respectively, *P* < 0.001). **VAS:** improvement in the VAS score in both groups but was greater in the Tr Gp; (27.19 versus 15.58 , *P* < 0.001) **Rate of collapse and THA:** – Tr Gp: 8 (21.05 %) hips collapsed and all 8 needed THA. – Ct Gp: 21 (45.65 %) hips collapsed and 19 (41.3%) needed THA (higher proportion in advanced stages)

**‘ACI’ Fontana *et al*. (2012): case control study** comparing simple arthroscopic debridement versus (ACT) for chondral lesions	– Patients with traumatic chondral lesions (Grade 3–4), mean size of lesion 2.6 cm^2^. – **Tr Gp:** 15 hips received arthroscopic ACT. – **Ct Gp:** 15 hips received arthroscopic debridement.	– Arthroscopic debridement and micro fracture for both groups. – Arthroscopic ACT for the treatment group using Autologous chondrocyte.	**FU:** average 74 months (72–76). **HHS:** improved in the both groups but more significantly in the Tr Gp (*P* < 0.001). – Mean HSS improvement for TR Gp and Ct Gp from 48.3 and 46 to 78.4 and 56.3, respectively.

### Bone morphogenetic proteins

Bone morphogenetic proteins (BMPs) are members of the TGF-β superfamily that are implicated in complex signalling pathways in osteoblastic differentiation and osteogenesis [2]. Hence they have been shown to possess oesteogenic activities and promote cartilage formation [2]. The efficacy of BMP-7 in the setting of bone non-union has been shown to be as effective as autologous bone graft [57]. Most of the studies we found were of case series describing the use of BMPs in the treatment of AVNFH.

Lieberman *et al*. [35] was the first to report on the use of BMP-7 in a case series of 17 hips (15 patients) with AVNFH (15 hips ficat stages IIA, one hip stage IIB and one stage III). Following core decompression and debridement of the necrotic lesion, autologous bone graft from the greater trochanter was impacted into the necrotic cavity with a gelatine capsule containing 50 mg of partially purified human BMP and non-collagenous protein. The decompression tract was filled with autolyzed cortical fibula bone graft. At the end of follow-up (mean duration of 53 months) there was no progression in 14 hips (86%), all from stage IIA. The other 3 hips had radiographic progression and all converted to total hip arthroplasty (THA). There was no comparative group and therefore the exact therapeutic impact of BMP on the overall outcome cannot be verified. The largest case series on the use of BMPs in AVNFH was published by Seyler *et al*. [36]. Their series included 39 hips, Ficat and Arlet Stage II or III. They used the trap door technique to make a window at the head–neck junction to remove the necrotic bone and to pack the excavated area with autologous cancellous bone graft, marrow and OP-1(BMP 7). The overall early clinical success (no THA) rate was 67% after a mean follow-up period of 36 month. The size of the lesion and the staging of AVNFH had a significant influence on the survival of the hips in their series.

The only comparative study found was a cohort study involving 72 hips and comparing the outcome of using artificial bone (Novobone) with or without rhBMP-2 for the treatment of AVNFH [19]. After a mean follow-up of 6 years, there was no statistically significant difference between the 2 groups in hip function score or radiological appearance of the femoral head (Table III).

One of the main complications associated with the use of BM-7 was highlighted by Papanagiotou *et al*. [37]. Heterotopic ossification developed in four of their seven patients but according to the authors this did not affect the post-operative rehabilitation or compromized the final results. The disease progressed in three of the seven patients following the treatment, two required THA (6 months after treatment).

### Platelet rich plasma and hyaluronic acid

A small number of studies have been published on the use of platelet rich plasma (PRP) in hip conservation surgery [20, 38–40, 45]. Only one comparative study was found assessing the effect of injecting PRP into the hip joint following arthroscopic surgery for labral tears [20]. After a follow-up period of 2 years the pain level in the PRP-treated patients was higher than the control group; however, there were no differences in hip function scores or rate of revision surgery (Table III).

A study concerning arthroscopic installation of PRP and bone grafting for the treatment AVNFH has been published recently [40]. Core decompression for grade I or IIA hips was achieved by drilling through the base of the head and then 10 ml of ‘liquid PRP’ was delivered into the necrotic area. In cases with advanced stage AVNFH (stage IIB and IIC), full debridement of the necrotic lesion was carried out by an arthroscopically created window in the head and neck junction. Autologous bone graft mixed with PRP was installed into the necrotic area. The authors described using this technique in three patients with a mean follow-up of 14 months. All three patients reported a significant reduction in pain intensity by >60% on a VAS scale and a return to activities of daily living by 5 months [40]. The use of PRP in combination with Adipose-tissue-derived stem cells (obtained from lipo-suction) and hyaluronic acid (HA) has also been described in three cases with early stages of AVNFH [38, 39]. This was injected into the femoral head under ultrasound guidance. The first two patients reported a reduction in pain by 70% and 30%, respectively, and filling of the bone defect 12 weeks following the procedure on MRI [39]. The third case [38] had further repeated injections of additional PRP into the osteonecrotic area on weekly bases for 4 weeks. Authors reported a complete resolution of the necrosis (clinically and radiologically) 21 months after the procedure but they had no biopsy to confirm.

### Synthetic bone substitutes

The majority of articles relating to bone substitutes reported their use as a mechanical support for the sub-chondral bone in AVNFH, building upon the use of autogenic and allogeneic bone graft. More recently, other support devices in the form of tantalum rods [15, 53], Nano-hydroxyapatite/polyamide 66 rod (n-HA/PA66) [18], porous calcium hydroxyapatite (IP-CHA) [11], injectable calcium sulphate (CaSO_4_)/calcium phosphate (CaPO_4_) composite graft [46] and calcium phosphate cement [47] have been used. Furthermore, the use of cell therapy in combination with the aforementioned materials has also been described in [11, 18].

Yang *et al.* [21] compared the outcome of using nano-hydroxyapatite/polyamide 66 rod (n-HA/PA66) and resorbable bioglass bone (NovaBone) graft versus autologous cancellous bone graft in patients with AVNFH. The group reported an improved outcome with regards to hip function score, pain and disease progression with the rod treatment after ∼2 years of follow-up (Table III). It should be noted that the paper was retrospective and that the techniques used for the debridement and graft impaction were different in the two groups.

In a case series, Civinini *et al*. [46] used an injectable calcium sulphate (CaSO_4_)/calcium phosphate (CaPO_4_) composite graft injected into the debrided necrotic cavity. This was augmented with autologous bone marrow concentrate to supplement the core decompression in the treatment of stages 1c–3a AVNFH in 37 hips. With a mean follow-up of 20 months (range 10–36 months), there was a significant increase in the Hip Harris Score (HHS) from a mean of 68–86 points (*P* < 0.05). Based on the HHS, 86.5% of patients had excellent or good outcome. There was radiological progression of the disease in 8 (21.6%) and clinical failure in 5(13.5%) patients (advanced stages), 3(8.1%) of whom needed THA.

Recently, a dynamic umbrella-shaped titanium alloy scaffold has been proposed for AVNFH [52]. This device is inserted via the decompression tunnel and implanted into the collapsed head. The umbrella is then expanded in the femoral head and the cavity is backed with autologous bone graft and artificial bone. Yu *et al*. [52] reported on the outcome of using these umbrella devices in 18 patients. After a mean follow-up time of 19 months, only 2 hips had a fair and one hip had a poor outcome. The rest had either good (9 hip) or excellent (5 hips) outcome. One patient had a major complication when the umbrella was misplaced and expanded outside the femoral head resulting in conversion to THA 10 days following the procedure.

Mini invasive arthroscopic grafting of bone cysts using cancellous bone and demineralized bone matrix has been described in two cases by Jamali *et al*. [48]. One patient had a cyst in the femoral neck and the other had a similar cyst in the acetabular rim. There was good filling of the defects and ‘excellent’ clinical outcome 20 and 6 months, respectively following the procedure. The authors recommended the graft material for this application to be osteoconductive, osteoinductive, malleable and is self-adherent to prevent fragment release into the joint risking third-body damage [48].

### Cell therapy

The use of cell therapy in the hip joint has only recently been explored and is still largely confined to the treatment of AVNFH, Table II. The push towards using cell therapy in the treatment of AVNFH came from the recognition that the replication capacity of osteoblastic cells and mesenchymal stem cells pool are significantly reduced in the proximal femur of patients with AVNFH [58, 59]. Hence supplementing the femoral head with viable multipotent cells could be considered as an appropriate pathophysiological approach for the treatment of this condition [60]. Pre-clinical studies have demonstrated survival of implanted cells in the necrotic region of the femur resulting in new bone formation and neovascularization [61].

The clinical use of Bone Marrow Mononuclear cells (BMMCs) in the treatment of AVNFH was initiated by Hernigou *et al*. in 2002 [62]. A recently published long-term outcome (follow-up ranging between 8 and 18 years) reported 17.6% conversion to THA. The rest (82.4%) had significant improvement in the HHS at the end of the follow-up [60]. They also reported a complete resolution of the disease in 12.9% of patients (all were ARCO stage 1) and a reduction in the necrotic lesion size in the rest of the hips (69.5%) [60]. The lack of a comparative (control) group in this large case series made it difficult to determine the exact effectiveness of cell therapy in AVNFH. However it demonstrated the feasibility of using cell therapy in the treatment of AVNFH, and the potential of achieving a better outcome relative to the traditional surgical treatments.

Over the last 12 years, several clinical control studies including five RCTs have been published reporting on the short-term outcome of incorporating multipotent cells with surgical treatment of early stages of AVNFH [11–18] (Table II). The mean duration of follow-up ranged from 24 months to 60 months and four of the five studies reported a better outcome in disease progression and clinical symptoms when cells were incorporated in the surgical treatment of AVNFH. These found that the time to progression and conversion to THA was also lower in patients treated with Cells [11–13, 15, 16, 18]. However based on the small number of studies, it is not clear whether this had an impact on the time delay to THA in failed cases [12, 61]. The largest therapeutic difference was observed in the earlier stages of AVNFH. The therapeutic difference decreased as the stages of the disease advanced (ARCO sage I to III); however, the overall effect of combining cell therapy in treating ARCO stage II and III appeared to be superior to the standard surgical treatment in the control groups [11–18].

Attempt to systemically summarize the results of all the published RCTs (or other control trials) to determine the overall combined effect of cell therapy has been challenging [61, 63]. This is because the studies have included cohort of patients with different aetiologies for the disease, used different classification systems for AVNFH and had different outcome measure scales and different endpoints (see Table II). A recent meta-analysis has attempted to analyse the results of four RCTs but could not combine all the studies into one analysis thus diluting the strength of the results [63].

Treatment of advanced stages of AVNFH with cells has been described by one case series. Zhao *et al*. [64] reported on the use of *ex vivo* expanded autologous BMMCs in the treatment of collapse or end stage (ARCOIIC-V) AVNFH. The cells were implanted in combination with a vascularized bone graft into the debrided necrotic area of the femoral head. A titanium rod was also used to augment this construct. They reported a high 5-year survival rate and a significant improvement in HHS in the surviving hips (from a mean of 39.84 to 79.11 points for ARCO stage IIIC hips, and from a mean of 37.0 to 74.25 points for stage IV hips). Disease progression occurred in only 15.8% (3 of the 19) of stage 3C hips and overall 5 of the 31 hips (16.1%) were converted to THA (2 stage IIIC hips and 3 stage IV hips). Although they did not have a control group, the results in this case series is better than that reported historically for vascularized graft alone [65]. However, the authors acknowledged this high success rate was confounded by the multiple procedures carried out in addition to the use of BMMCs. Hence it’s difficult to define the exact therapeutic contribution of BMMCs to the overall outcome.

In AVNFH, the BMMCs are implanted directly into the necrotic area of the femoral head. Tracking studies of these cells in patients showed 56% of installed cells remained in the implantation site 24 h after implantation [12]. Tracking studies in animals also demonstrated the survival and multiplications of these cells 12 weeks post implantation [66]. Other surgeons however, have used targeted infusion of peripheral blood stems cells (PBSCs) [15] and BMMCs [67] through the medial circumflex artery. Cells have been tracked to the necrotic femoral head following intravenous infusion in animal models [68], but this has not been verified in human patients [15, 67], and this therefore might have an implication on the quantities of cells reaching and residing in the necrotic area. This is important because Hernigou *et al*. [60] reported an association between the outcome of AVNFH and the quantity of cells transplanted into the femoral head and recommended a specific minimum number of oestrogenic precursor cells transplantation [14, 69]. No studies have compared directly implanted cells into the necrotic area with cells indirectly transplanted via targeted arterial infusion. However direct comparison between the use of concentrated BMMCs versus unprocessed bone marrow aspirate has shown a better clinical outcome with processed (concentrated) BMMCs [17], yielding further support for the importance of high BMMCs concentrate transplantation. This may also highlight the importance of *ex* *vivo* cells expansion (prior transplantation) in patients who have low BMMCs concentration (such as in cases of steroid and alcohol induced AVNFH [70]) in order to overcome the inconsistencies in the number of cells transplanted [13, 60, 71]. This literature search was unable to identify control studies comparing the outcome of *ex* *vivo* expanded cells with processed and concentrated BMMCs directly from bone marrow aspirate.

In the studies reviewed, no serious adverse events in association with the use of cell therapy in the treatment of AVNFH (especially with regards to malignancy) have been reported. However, it must be stressed that only the short- and the mid-term results have been published so far.

### Autologous chondrocyte implantation

Autologous chondrocyte implantation (ACI) involves initial debridement and harvest followed by transplantation of culture expanded chondrocytes into the chondral defect. Chondrocytes are either sealed with periosteum or are delivered bound to a layer of collagen-matrix [22, 41, 42, 56].

The first case report of using ACI for the treatment of a femoral head articular defect was published by Akimau *et al*. [44]. Following anterior dislocation of the hip joint, the defect was debrided, filled with bone graft and sealed with a collagen patch. Six million chondrocytes were injected under the patch. The patient’s HSS improved from 52 (pre-operatively) to 76 points, 16 months following the procedure, and was able to run and walk more than a mile. Full depth histological biopsy from the ACI treated site 15 months post-surgery demonstrated predominantly fibrocartilage of ∼2-mm thickness, fully integrated with the underlying bone and contained viable cells.

The largest case series of arthroscopic autologous chondrocyte transplantation in the hip joint was reported by Körsmeier *et al*. [42]. Autologous matrix-induced 3D chondrocyte transplantation spheroids (ACT 3D) were used to treat 16 hips with acetabular chondral defects induced by CAM-type femoroacetabular impingement (FAI). The cartilage was harvested from the cam lesion and sent to an offsite centre for chondrocyte culture into 3D spheroids over a period of 5–10 weeks (each spheroid contained 200 000 chondrocytes). A second stage arthroscopy was carried out to debride the defect and deliver the spheroids using a flexible needle. The average size of the chondral defects was 4.52 cm^2^ (range 3–6 cm^2^). There was a significant improvement in the WOMAC and NAHS 6 and 12 weeks after grafting (*P* < 0.001 and < 0.001, respectively). These scores were maintained after a mean follow-up period of 16.09 months (range 9.5–28.8 months). The outcome of surgery with regard to pain relief, restoration of mobility and ability to perform sporting activities was reported by patients as excellent in eight cases, very good in four, good in three and fair in one case [42].

There is only one comparison study involving ACI, published by Fontana *et al.* [22]. They retrospectively compared the outcome of ACI to simple microfracture in patients who had third or fourth degree (Outerbridge classification) chondral lesions of 2 cm^2^ or more. An initial arthroscopy was carried out to grade and debride the lesion and to obtain a cartilage biopsy from the pulvinar. The cells were expanded and subsequently implanted using a 3D polymer scaffold during a second arthroscopic procedure. The mean follow-up was ∼6 years for both groups (73.8 months in the ACI group and 74.3 months in the debridement group). There was a statistically significant difference in the mean HHS at the end of the follow-up between the two groups (*P* < 0.001) (Table III). No post-procedure radiological or arthroscopic evaluations for these patients were carried out.

### Mosaicplasty and osteochondral transplantation

Although mosaicplasty and osteochondral transplant (OT) do not fit the definition of an ‘orthobiologic’ but were identified in the search strategy and are included here for completeness and relevance to the emerging field of hip preservation.

OT in the femoral head and the acetabular dome has been reported in various case reports and case series [23–33, 49–51]. Authors recommended OT for small articular cartilage defects with stable and well perfused sub-chondral bone surrounded by a stable bed of cartilage [72]. Mosaicplasty involves the use of multiple small osteochondral plugs to treat larger lesions. The articles identify that it is important to restore the original geometry and curvature of the femoral head or acetabular surface [27, 72]. Therefore grafts are ideally harvested from a donor source that resembles the original curvature of the defective articular surface. Hence, many harvest the autologous graft from the non weight bearing portion of the femoral head or an allograft from the same corresponding area on the femoral head or the acetabular dome. Similarly, mosaicplasty plugs harvested from the knee joint are arranged in a way to restore the natural curvature of the femoral head [27].

Kosashvili *et al.* [25] published a case series of eight patients who received OT for chondral lesions in the femoral head. The procedure involved trochanteric osteotomy and open dislocation of the hip followed by debridement and reaming of the osteochondral defect. Matching size fresh-stored osteochondral allograft was implanted using a press fit technique. Follow-up ranged from 24 to 54 months (mean; 41 months). The mean HHS improved from 57.7 (range 50–65) points pre-operatively to 83.9 (range 72–94) points at the latest follow-up. The procedure failed in two patients, one of whom needed THA 6 month after the procedure. The failure in the second patient was attributed to non-compliance with the rehab protocol resulting in subsidence of the graft. Hence a revision osteochondral allograft transfer was performed.

The largest case series with the longest follow-up for OT has been reported by Mayers *et al*. [28]. They used allogeneic osteohondral graft to treat 25 hips, all (except one) had segmental collapse of the femoral head secondary to AVNFH. They reported a 32% failure rate over a follow-up period ranging between 9 and 63 months. In total 50% of failures occurred in patients with AVNFH secondary to fractured neck of femur or steroids, all occurring within the first 18 months after treatment.

For femoral head mosaicplasty, the largest case series was published by Girard *et al*. [27]. They reported the clinical outcome of treating 10 patients by open anterior dislocation of the femoral head and mosaicplasty. The size of the lesions ranged from 3 to 9 cm^2^ (mean 4.8 cm^2^). Osteochondral plugs (8–10 mm) were harvested from the non-weight bearing portion of the femoral head. After a mean follow-up period of 29.2 (range, 20–33) months there was an improvement in the Postel Merle d’Aubigne score and the HSS from 10.5 and 52.8 pre-operatively, to 15.5 and 79.5, respectively. There was also a decrease in the Oxford Hip Score indicating improvement in function 34.5 (22–48) to 19.2 (14–26) and improvement in patients activity of daily living. Radiological assessment revealed an excellent incorporation of the graft and smooth articulating surface with no evidence of collapse.

The treatment of chondral defects in the acetabula dome using osteochondral allograft has so far been described in two reports [26, 33]. Krych *et al*. [26] used fresh stored, non-irradiated osteochondral allografts that were press fitted into the defects without supplementary fixation. MRI scans at 12 and 18 months demonstrated incorporation of the osteochondral allograft to the host bone with maintenance of joint congruity. The two patients had HSS of 97 and 100 at the end of 2 and 3 years follow-up, respectively.

A technique for arthroscopic assisted autologous OT for the femoral head has also been reported [31]. In this procedure, a tunnel is created from the lateral cortex of the femur via the femoral neck and into the chondral defect. Reporting on two patients, the autologous osteochondral plug was harvested from the knee joint and passed retrogradely through the femoral neck tunnel into the chondral defect in the femoral head. After at least 2-year follow-up, the HSS improved from 56.6 and 88.6 pre-operatively, to 87.6 and 90, respectively. Neither of the patients had significant symptoms at the last follow-up.

### Other non-invasive hip preservation procedures

#### Autologous matrix-induced chondrogenesis

Autologous matrix-induced chondrogenesis (AMIC) is a procedure that involves microfracture of the sub-chondral bone and a type I/III collagen matrix is placed over the defect to provide a ceiling for and to support the haematoma that arises from microfracture [56]. It also provides a skeleton for repair and tissue formation. AMIC is carried out as a single stage procedure and does not involve implantation of chondrocytes which distinguishes it from ACI and MACI. The procedure is indicated for grade III or IV chondral lesions (Outerbridge classification) with sizes between 2 and 8 cm^2^. Fontana *et al.* [56] described arthroscopic AMIC for chondral lesions in the hip joint but so far there have been no published clinical outcome results of hips treated with this procedure.

#### Delaminated articular cartilage repair

Stafford *et al*. [54] reported on the arthroscopic repair of fully ‘enclosed’ delaminated acetabular cartilage using fibrin adhesive glue. The process involves microfracture of the sub-chondral bone behind the delaminated cartilage and subsequent injection of the fibrin adhesive into the cartilage pocket. The delaminated chondral surface is then firmly pressed against the chondral bone for 2 min. The outcome of this technique was reported in 43 patients who had an underpinning CAM lesion causing the delamination of the cartilage. After a mean follow-up of 28 months, there was a significant improvement in MHHS from 61.9 points to 79.4 and a significant improvement in MHHS for pain (*P* < 0.001 *P* = 0.006, respectively). Arthroscopic review of the repair in three patients (during a late arthroscopic iliopsoas release) demonstrated good repair of the chondral defects.

Sekiya *et al*. [55] described a suture repair of 1-cm delaminated cartilage flap in the anterior-superior acetabulum in a young patient with FAI. They carried out a microfracture of the sub-chondral bone underneath the flap before suturing it with an absorbable polydiaxanone monofilament. The patient reported 95% of normal function after 2 years of follow-up and good hip function scores (MHHS of 96, HOSADL of 93 and HOSSSS of 81).

## CONCLUSION

Orthobiologics is a difficult term to accurately define. We have used a systematic search of the literature to identify potentially relevant topics and then selected those most applicable to hip preservation surgery. In terms of the topics, we identified several innovative strategies that span disciplines from cell therapy to material approaches. However, the majority of the literature identified addressed the treatment of AVN of the femoral head which represent only a small proportion of hip preservation surgery. Furthermore, it is not clear to the authors that the term orthobiologics represents a useful term when compared with other similar and overlapping terms e.g. regenerative medicine, which has a more precise but also more focused definition.

Although the identified treatments may offer enormous potential for the future, when it comes to current practice the situation is not clear. The literature is dominated by level 3 and 4 evidence mainly in the form of short-term outcome results. There was a lack of comparative clinical trial data to inform evidence-based practice. Similar strategies are more clearly understood in the knee and it would seem appropriate to infer that they would be similarly useful in the hip, with supporting evidence. Hence there is an urgent need for better well constructed studies to establish the effectiveness of these orthobiologics and to refine their application in hip preservation surgery.

Finally, the exciting field of orthobiologics in joint preservation procedures brings with it regulatory and safety issues that will need to be successfully addressed. In this review, none of the studies reported any major adverse events but the quality of the evidence remains inadequate with long term safety data still required.

In summary orthobiologics, as set out in this account, is an overarching term for many approaches that offer a new and exciting direction for orthopaedic surgery in general and hip preservation specifically. However, the innovation must be carefully adopted by responsible translation. This will require robust clinical trial data to support both effectiveness and cost-effectiveness and needs to be underpinned by appropriate regulatory and safety data.

## CONFLICT OF INTEREST STATEMENT

None of the authors has received funding or any form of support for the work or the preparation of this manuscript. However support relating directly or indirectly to the subject of this article have been or will be received solely for research, education, or other non- profit organization with which one or more of the authors are associated.

## References

[1] AAOS website. Helping Fractures Heal (Orthobiologics). Available at: http://orthoinfo.aaos.org/topic.cfm?topic=A00525. Accessed: 2 June 2015.

[2] Roberts TT Rosenbaum AJ Bone grafts, bone substitutes and orthobiologics: the bridge between basic science and clinical advancements in fracture healing. Organogenesis 2012; 8: 114–24. 23247591 10.4161/org.23306 PMC3562252

[3] Dhillon RS Schwarz EM Maloney MD Platelet-rich plasma therapy—future or trend? *Arthritis Res* Ther 2012; 14: 219. 22894643 10.1186/ar3914 PMC3580559

[4] Zgonis T Current update on orthobiologics in foot and ankle surgery. Clin Podiatr Med Surg 2015; 32: xv. 25440426 10.1016/j.cpm.2014.10.002

[5] Singh K Nandyala SV Marquez-Lara A Fineberg SJ Epidemiological trends in the utilization of bone morphogenetic protein in spinal fusions from 2002 to 2011. Spine (Phila Pa 1976) 2014; 39: 491–6. 24365905 10.1097/BRS.0000000000000167

[6] Saw KY Anz A Merican S Articular cartilage regeneration with autologous peripheral blood progenitor cells and hyaluronic acid after arthroscopic subchondral drilling: a report of 5 cases with histology. Arthroscopy 2011; 27: 493–506. 21334844 10.1016/j.arthro.2010.11.054

[7] Dold AP Zywiel MG Taylor DW Dwyer T Theodoropoulos J Platelet-rich plasma in the management of articular cartilage pathology: a systematic review. Clin J Sport Med 2014; 24: 31–43. 24231930 10.1097/01.jsm.0000432855.85143.e5

[8] Saw KY Anz A Siew-Yoke Jee C Articular cartilage regeneration with autologous peripheral blood stem cells versus hyaluronic acid: a randomized controlled trial. Arthroscopy 2013; 29: 684–94. 23380230 10.1016/j.arthro.2012.12.008

[9] Khanduja V Villar RN Arthroscopic surgery of the hip: current concepts and recent advances. J Bone Joint Surg Br 2006; 88: 1557–66. 17159164 10.1302/0301-620X.88B12.18584

[10] Shetty VD Villar RN Hip arthroscopy: current concepts and review of literature. Br J Sports Med 2007; 41: 64–8; discussion 68. 17138638 10.1136/bjsm.2006.027755 PMC2658928

[11] Yamasaki T Yasunaga Y Ishikawa M Bone-marrow-derived mononuclear cells with a porous hydroxyapatite scaffold for the treatment of osteonecrosis of the femoral head: a preliminary study. J Bone Joint Surg Br 2010; 92: 337–41. 20190302 10.1302/0301-620X.92B3.22483

[12] Gangji V De Maertelaer V Hauzeur JP Autologous bone marrow cell implantation in the treatment of non-traumatic osteonecrosis of the femoral head: five year follow-up of a prospective controlled study. Bone 2011; 49: 1005–9. 21821156 10.1016/j.bone.2011.07.032

[13] Zhao D Cui D Wang B Treatment of early stage osteonecrosis of the femoral head with autologous implantation of bone marrow-derived and cultured mesenchymal stem cells. Bone 2012; 50: 325–30. 22094904 10.1016/j.bone.2011.11.002

[14] Sen RK Tripathy SK Aggarwal S Early results of core decompression and autologous bone marrow mononuclear cells instillation in femoral head osteonecrosis: a randomized control study. J Arthroplasty 2012; 27: 679–86. 22000577 10.1016/j.arth.2011.08.008

[15] Mao Q Wang W Xu T Combination treatment of biomechanical support and targeted intra-arterial infusion of peripheral blood stem cells mobilized by granulocyte-colony stimulating factor for the osteonecrosis of the femoral head: a randomised controlled clinical trial. J Bone Miner Res 2014; 30: 647–56. 25349059 10.1002/jbmr.2390 PMC4376653

[16] Ma Y Wang T Liao J Efficacy of autologous bone marrow buffy coat grafting combined with core decompression in patients with avascular necrosis of femoral head: a prospective, double-blinded, randomized, controlled study. Stem Cell Res Ther 2014; 5: 115. 25315149 10.1186/scrt505 PMC4446117

[17] Rastogi S Sankineani SR Nag HL Intralesional autologous mesenchymal stem cells in management of osteonecrosis of femur: a preliminary study. Musculoskelet Surg 2013; 97: 223–8. 23852661 10.1007/s12306-013-0273-0

[18] Liu Y Liu S Su X Core decompression and implantation of bone marrow mononuclear cells with porous hydroxylapatite composite filler for the treatment of osteonecrosis of the femoral head. Arch Orthop Trauma Surg 2013; 133: 125–33. 23070224 10.1007/s00402-012-1623-3

[19] Sun W Li Z Gao F Recombinant human bone morphogenetic protein-2 in debridement and impacted bone graft for the treatment of femoral head osteonecrosis. PLoS One 2014; 9: e100424. 24956102 10.1371/journal.pone.0100424 PMC4067369

[20] Redmond JM Gupta A Stake CE Clinical results of hip arthroscopy for labral tears: a comparison between intraoperative platelet-rich plasma and bupivacaine injection. Arthroscopy 2014; 31: 445–53. 25442663 10.1016/j.arthro.2014.08.034

[21] Yang P Bian C Huang X Core decompression in combination with nano-hydroxyapatite/polyamide 66 rod for the treatment of osteonecrosis of the femoral head. Arch Orthop Trauma Surg 2014; 134: 103–12. 24248422 10.1007/s00402-013-1885-4

[22] Fontana A Bistolfi A Crova M Arthroscopic treatment of hip chondral defects: autologous chondrocyte transplantation versus simple debridement—a pilot study. Arthroscopy 2012; 28: 322–9. 22142720 10.1016/j.arthro.2011.08.304

[23] Sotereanos NG DeMeo PJ Hughes TB Autogenous osteochondral transfer in the femoral head after osteonecrosis. Orthopedics 2008; 31: 177. 19292191 10.3928/01477447-20080201-33

[24] Nam D Shindle MK Buly RL Traumatic osteochondral injury of the femoral head treated by mosaicplasty: a report of two cases. HSS J 2010; 6: 228–34. 21886541 10.1007/s11420-010-9159-y PMC2926357

[25] Kosashvili Y Raz G Backstein D Fresh-stored osteochondral allografts for the treatment of femoral head defects: surgical technique and preliminary results. Int Orthop 2013; 37: 1001–6. 23553116 10.1007/s00264-013-1868-7 PMC3664145

[26] Krych AJ Lorich DG Kelly BT Treatment of focal osteochondral defects of the acetabulum with osteochondral allograft transplantation. Orthopedics 2011; 34: e307–11. 21717995 10.3928/01477447-20110526-24

[27] Girard J Roumazeille T Sakr M Migaud H Osteochondral mosaicplasty of the femoral head. Hip Int 2011; 21: 542–8. 21948031 10.5301/HIP.2011.8659

[28] Meyers MH Resurfacing of the femoral head with fresh osteochondral allografts. Long-term results. Clin Orthop Relat Res 1985; 197: 111–4. 3893823

[29] Meyers MH The surgical treatment of osteonecrosis of the femoral head with an osteochondral allograft. *Acta Orthop Belg*. 1999; 65(Suppl 1): 66–7. 10084220

[30] Hart R Janecek M Visna P Mosaicplasty for the treatment of femoral head defect after incorrect resorbable screw insertion. Arthroscopy 2003; 19: E1–5. 14673462 10.1016/j.arthro.2003.10.025

[31] Cetinkaya S Toker B Taser O Arthroscopic retrograde osteochondral autologous transplantation to chondral lesion in femoral head. Orthopedics 2014; 37: e600–4. 24972445 10.3928/01477447-20140528-64

[32] Emre TY Cift H Seyhan B Mosaicplasty for the treatment of the osteochondral lesion in the femoral head. Bull NYU Hosp Joint Dis 2012; 70: 288–90. 23267459

[33] Field RE Rajakulendran K Strambi F Arthroscopic grafting of chondral defects and subchondral cysts of the acetabulum. Hip Int 2011; 21: 479–86. 21818747 10.5301/HIP.2011.8583

[34] Calori GM Mazza E Colombo M Treatment of AVN using the induction chamber technique and a biological-based approach: indications and clinical results. Injury 2014; 45: 369–73. 24119830 10.1016/j.injury.2013.09.014

[35] Lieberman JR Conduah A Urist MR Treatment of osteonecrosis of the femoral head with core decompression and human bone morphogenetic protein. Clin Orthop Relat Res 2004; 429: 139–45. 15577478 10.1097/01.blo.0000150312.53937.6f

[36] Seyler TM Marker DR Ulrich SD Nonvascularized bone grafting defers joint arthroplasty in hip osteonecrosis. Clin Orthop Relat Res 2008; 466: 1125–32. 18351424 10.1007/s11999-008-0211-x PMC2311481

[37] Papanagiotou M Malizos KN Vlychou M Dailiana ZH Autologous (non-vascularised) fibular grafting with recombinant bone morphogenetic protein-7 for the treatment of femoral head osteonecrosis: preliminary report. Bone Joint J 2014; 96-B: 31–5. 24395307 10.1302/0301-620X.96B1.32773

[38] Pak J Lee JH Jeon JH Lee SH Complete resolution of avascular necrosis of the human femoral head treated with adipose tissue-derived stem cells and platelet-rich plasma. J Int Med Res 2014; 42: 1353–62. 25281062 10.1177/0300060514546940

[39] Pak J Regeneration of human bones in hip osteonecrosis and human cartilage in knee osteoarthritis with autologous adipose-tissue-derived stem cells: a case series. J Med Case Rep 2011; 5: 296. 21736710 10.1186/1752-1947-5-296 PMC3154169

[40] Guadilla J Fiz N Andia I Sanchez M Arthroscopic management and platelet-rich plasma therapy for avascular necrosis of the hip. Knee Surg Sports Traumatol Arthrosc 2012; 20: 393–8. 21695463 10.1007/s00167-011-1587-9

[41] Ellender P Minas T Autologous chondrocyte implantation in the hip: case report and technique. Oper Tech Sports Med 2008; 16: 201–6.

[42] Korsmeier K Classen T Kamminga M Arthroscopic three-dimensional autologous chondrocyte transplantation using spheroids for the treatment of full-thickness cartilage defects of the hip joint. Knee Surg Sports Traumatol Arthrosc 2014. 10.1007/s00167-014-3293-x 25223968

[43] Fickert S Schattenberg T Niks M Feasibility of arthroscopic 3-dimensional, purely autologous chondrocyte transplantation for chondral defects of the hip: a case series. Arch Orthop Trauma Surg 2014; 134: 971–8. 24777539 10.1007/s00402-014-1997-5

[44] Akimau P Bhosale A Harrison PE Autologous chondrocyte implantation with bone grafting for osteochondral defect due to posttraumatic osteonecrosis of the hip—a case report. Acta Orthop 2006; 77: 333–6. 16752299 10.1080/17453670610046208

[45] Martin JR Houdek MT Sierra RJ Use of concentrated bone marrow aspirate and platelet rich plasma during minimally invasive decompression of the femoral head in the treatment of osteonecrosis. Croat Med J 2013; 54: 219–24. 23771751 10.3325/cmj.2013.54.219 PMC3692329

[46] Civinini R De Biase P Carulli C The use of an injectable calcium sulphate/calcium phosphate bioceramic in the treatment of osteonecrosis of the femoral head. Int Orthop 2012; 36: 1583–8. 22426934 10.1007/s00264-012-1525-6 PMC3535041

[47] Rijnen WH Gardeniers JW Schreurs BW Buma P Impacted bone and calcium phosphate cement for repair of femoral head defects: a pilot study. Clin Orthop Relat Res 2007; 459: 216–21. 17308484 10.1097/BLO.0b013e3180373138

[48] Jamali AA Fritz AT Reddy D Meehan JP Minimally invasive bone grafting of cysts of the femoral head and acetabulum in femoroacetabular impingement: arthroscopic technique and case presentation. Arthroscopy 2010; 26: 279–85. 20141992 10.1016/j.arthro.2009.09.016

[49] Gagala J Tarczynska M Gaweda K Clinical and radiological outcomes of treatment of avascular necrosis of the femoral head using autologous osteochondral transfer (mosaicplasty): preliminary report. Int Orthop 2013; 37: 1239–44. 23632987 10.1007/s00264-013-1893-6 PMC3685652

[50] Evans KN Providence BC Case report: Fresh-stored osteochondral allograft for treatment of osteochondritis dissecans the femoral head. Clin Orthop Relat Res 2010; 468: 613–8. 19727986 10.1007/s11999-009-0997-1 PMC2806996

[51] Philippon MJ Jarvis HC Arthroscopic management of a femoral head osteochondral defect using autologous osteochondral transfer, platelet-rich plasma and microfracture. *Cur Ortho Prac*t 2012; 23: 629–33.

[52] Yu X Jiang W Pan Q Umbrella-shaped, memory alloy femoral head support device for treatment of avascular osteonecrosis of the femoral head. Int Orthop 2013; 37: 1225–32. 23532589 10.1007/s00264-013-1869-6 PMC3685662

[53] Zhao D Zhang Y Wang W Tantalum rod implantation and vascularized iliac grafting for osteonecrosis of the femoral head. Orthopedics 2013; 36: 789–95. 23746017 10.3928/01477447-20130523-26

[54] Stafford GH Bunn JR Villar RN Arthroscopic repair of delaminated acetabular articular cartilage using fibrin adhesive. Results at one to three years. Hip Int 2011; 21: 744–50. 22117261 10.5301/HIP.2011.8843

[55] Sekiya JK Martin RL Lesniak BP Arthroscopic repair of delaminated acetabular articular cartilage in femoroacetabular impingement. Orthopedics 2009; 32: pii. 10.3928/01477447-20090728-44 19750994

[56] Fontana A A novel technique for treating cartilage defects in the hip: a fully arthroscopic approach to using autologous matrix-induced chondrogenesis. Arthrosc Tech 2012; 1: e63–8. 23766978 10.1016/j.eats.2012.02.003 PMC3678655

[57] White AP Vaccaro AR Hall JA Clinical applications of BMP-7/OP-1 in fractures, nonunions and spinal fusion. Int Orthop 2007; 31: 735–41. 17962946 10.1007/s00264-007-0422-x PMC2266670

[58] Gangji V Hauzeur JP Schoutens A Abnormalities in the replicative capacity of osteoblastic cells in the proximal femur of patients with osteonecrosis of the femoral head. J Rheumatol 2003; 30: 348–51. 12563694

[59] Hernigou P Beaujean F Lambotte JC Decrease in the mesenchymal stem-cell pool in the proximal femur in corticosteroid-induced osteonecrosis. J Bone Joint Surg Br 1999; 81: 349–55. 10204950 10.1302/0301-620x.81b2.8818

[60] Hernigou P Poignard A Zilber S Rouard H Cell therapy of hip osteonecrosis with autologous bone marrow grafting. Indian J Orthop 2009; 43: 40–5. 19753178 10.4103/0019-5413.45322 PMC2739495

[61] Lau RL Perruccio AV Evans HM Stem cell therapy for the treatment of early stage avascular necrosis of the femoral head: a systematic review. BMC Musculoskelet Disord 2014; 15: 156. 24886648 10.1186/1471-2474-15-156 PMC4038713

[62] Hernigou P Beaujean F Treatment of osteonecrosis with autologous bone marrow grafting. Clin Orthop Relat Res 2002; 405: 14–23. 12461352 10.1097/00003086-200212000-00003

[63] Li X Xu X Wu W Comparison of bone marrow mesenchymal stem cells and core decompression in treatment of osteonecrosis of the femoral head: a meta-analysis. Int J Clin Exp Pathol 2014; 7: 5024–30. 25197374 PMC4152064

[64] Zhao D Liu B Wang B Autologous bone marrow mesenchymal stem cells associated with tantalum rod implantation and vascularized iliac grafting for the treatment of end-stage osteonecrosis of the femoral head. BioMed Res Int 2014; 2014: 240506. 10.1155/2015/240506 PMC4352743 25802840

[65] Zhao D Xu D Wang W Cui X Iliac graft vascularization for femoral head osteonecrosis. Clin Orthop Relat Res 2006; 442: 171–9. 16394757 10.1097/01.blo.0000181490.31424.96

[66] Yan Z Hang D Guo C Chen Z Fate of mesenchymal stem cells transplanted to osteonecrosis of femoral head. J Orthop Res 2009; 27: 442–6. 18925660 10.1002/jor.20759

[67] Mao Q Jin H Liao F The efficacy of targeted intraarterial delivery of concentrated autologous bone marrow containing mononuclear cells in the treatment of osteonecrosis of the femoral head: a five year follow-up study. Bone 2013; 57: 509–16. 23994171 10.1016/j.bone.2013.08.022 PMC3927161

[68] Li ZH Liao W Cui XL Intravenous transplantation of allogeneic bone marrow mesenchymal stem cells and its directional migration to the necrotic femoral head. Int J Med Sci 2011; 8: 74–83. 21234272 10.7150/ijms.8.74 PMC3020395

[69] Hernigou P Manicom O Poignard A Core decompression with marrow stem cells. Oper Tech Orthop 2004; 14: 68–74.

[70] Hernigou P Beaujean F Abnormalities in the bone marrow of the iliac crest in patients who have osteonecrosis secondary to corticosteroid therapy or alcohol abuse. J Bone Joint Surg Am 1997; 79: 1047–53. 9234881 10.2106/00004623-199707000-00011

[71] Aoyama T Goto K Kakinoki R An exploratory clinical trial for idiopathic osteonecrosis of femoral head by cultured autologous multipotent mesenchymal stromal cells augmented with vascularized bone grafts. Tissue Eng Part B Rev 2014; 20: 233–42. 24593258 10.1089/ten.teb.2014.0090 PMC4123560

[72] Jordan MA Van Thiel GS Chahal J Nho SJ Operative treatment of chondral defects in the hip joint: a systematic review. Curr Rev Musculoskelet Med 2012; 5: 244–53. 22814674 10.1007/s12178-012-9134-y PMC3535089

